# Thinning of maximum ciliary body thickness: a potential early indicator for pseudophakic malignant glaucoma in primary angle closure glaucoma

**DOI:** 10.1186/s12886-025-04100-0

**Published:** 2025-04-28

**Authors:** Yong Jie Qin, Fu Long Luo, Jin Zeng, Yu Lin Zhang, Wen Juan Xie, Yan Lei Chen, Sun On Chan, Hong Yang Zhang

**Affiliations:** 1 Department of Ophthalmology, Nanfang Hospital, Southern Medical University, Guangzhou, China; 2 Department of Ophthalmology, Guangdong Provincial People’s Hospital (Guangdong Academy of Medical Sciences), Southern Medical University, Guangzhou, China; 3 School of Biomedical Sciences, The Chinese University of Hong Kong, Hong Kong, China; 4 Guangdong Cardiovascular Institute, Guangdong Provincial People’s Hospital, Guangdong Academy of Medical Sciences, Guangzhou, China

**Keywords:** Pseudophakic malignant glaucoma, Ciliary body thickness, Ultrasound biomicroscopy

## Abstract

**Objective:**

Pseudophakic malignant glaucoma (PMG) is an uncommon but severe postoperative complication that poses a significant threat to vision. Early detection of PMG in patients with primary angle closure glaucoma (PACG) is imperative for effective intervention. This study sought to determine whether specific morphometric indicators could predict the onset of PMG.

**Methods:**

A retrospective cross-sectional analysis was performed on data collected from June 2016 to May 2023. The study population comprised PACG patients who developed PMG after phacoemulsification, with a control group of eyes that did not. Ultrasound biomicroscopy (UBM) was employed to measure the central anterior chamber depth (ACD), trabecular-ciliary process angle (TCA), and ciliary body thickness at multiple points (CBTmax, CBT0, CBT1000), as well as the anterior placement of the ciliary body (APCB). These measurements were taken at three distinct phases: pre-onset, onset, and 6 months following PMG resolution.

**Results:**

The study encompassed 60 eyes from 60 patients, with baseline characteristics showing no significant differences between the groups. Following lens extraction, a notable increase in CBTmax, CBT0, and TCA was observed in matched eyes, but not detected in those that developed PMG. At pre-onset of PMG, a significant reduction in CBTmax was identified exclusively in eyes that later exhibited PMG (0.87 ± 0.09 mm vs. 0.95 ± 0.09 mm, *P* = 0.001), when compared to the matched eyes. The resolution of PMG through zonulo-hyaloido-vitrectomy was associated with an increase in ACD, CBTmax, CBT0, and TCA. Notably, the pre-onset CBTmax was the sole parameter to exhibit significant prognostic value for PMG development (0.74 [95% CI, 0.61–0.87], *P* = 0.001), nearly matching the predictive accuracy during PMG attack (0.86 [95% CI, 0.76–0.96], *P* < 0.001).

**Conclusion:**

A reduction in ciliary body thickness, particularly CBTmax, appears to be a pre-existing condition in eyes that develop PMG from PACG. This parameter holds promise as a sensitive early predictor, potentially improving the timeliness of PMG diagnosis and treatment.

**Supplementary Information:**

The online version contains supplementary material available at 10.1186/s12886-025-04100-0.

## Introduction

Malignant glaucoma is a rare but vision-threatening condition that is characterized by the forward displacement of iris-lens diaphragm leading to persistent flattening of the anterior chamber. Most cases develop after glaucoma surgery [1], but some may occur after lens extraction [2, 3]. In general, aphakic or pseudophakic eyes are unsusceptible to developing malignant glaucoma because lens removal effectively increases the depth of anterior chamber [4]. Thus, the term malignant glaucoma may not be applied to the glaucoma that occurs in pseudophakia [5]. However, increasing cases of this syndrome were reported in a number of studies [6–8]. The precise pathology of why malignant glaucoma develops in the eyes without the crystalline lens remains unclear. A short axial length (AL) and a narrow anterior chamber depth (ACD) were reported consistently in the eyes with pseudophakic malignant glaucoma [7]. In patients with shorter AL and shallower ACD, malignant glaucoma should be alerted after cataract surgery.

However, we currently found that after lens extraction, malignant glaucoma was observed in some patients, but was absent in the others, even when these patients have similar ACD and AL [9]. It suggests that in addition to ACD and AL, other factors should be considered that may associate with the development of malignant glaucoma. Previously, inflammation, zonular weakness and intact anterior hyaloid were proposed as triggering factors causing irido-cilio-vitreal block in aphakic or pseudophakic eyes [10, 11]. However, these factors are difficult to quantify in patients. With ultrasound biomicroscopy (UBM), swelling and anterior rotation of the ciliary processes can be readily visualized, which are reported as major causes of ciliary block [2, 12]. While UBM was commonly performed during malignant glaucoma attack, it did not equally reflect the condition of a resting ciliary body. Notably, the structure of ciliary body could have been affected by treatment with cycloplegics, anti-inflammatory drugs, and pressure-lowering agents [13]. Thus, if measurement is done before the malignant glaucoma attack, it would be more reasonable and reliable to identify the risk factors for early prediction. We therefore reviewed 60 patients who had UBM done before the onset of malignant glaucoma and found that a reduction of maximum ciliary body thickness (CBTmax) might serve as a reliable pre-existing predictor for pseudophakic malignant glaucoma.

## Methods

### Study population

This retrospective study was approved by the institutional Human Research Ethics Committee of Guangdong Provincial People’s Hospital, Guangzhou, China, and adhered to the tenets of the Declaration of Helsinki. Informed consent was obtained from each patient upon the final follow-up. A total of 60 eyes from 60 patients were recruited between June 2016 and May 2023. They were diagnosed originally with primary angle-closure glaucoma (PACG) and received an uneventful phacoemulsification with in-the-bag intraocular lens (IOL) placement. The onset of malignant glaucoma was defined as the time after phacoemulsification when the diagnosis of malignant glaucoma was confirmed by experienced glaucoma specialists (HYZ and YJQ). Alternate causes for anterior chamber shallowness, such as pupillary block, capsule block syndrome with retained ophthalmic viscosurgical device behind the IOL, posterior segment mass, suprachoroidal hemorrhage, ciliary body effusion, plateau iris or pseudoplateau phenomenon, and pseudophacodonesis were excluded from the current study. Patients who had combined glaucoma and cataract surgery were also excluded. Patients without occurrence of malignant glaucoma after cataract surgery were recruited as matched eyes. All subjects underwent detailed ocular examinations, including best-corrected visual acuity (BCVA), slit-lamp examination, IOP measurement by Goldmann applanation tonometry, and stereoscopic optic disc examination with a 90-diopter lens. Gonioscopy was performed in the dark using Goldmann 1-mirror lens (Ocular Instruments., Bellevue, WA) and the anterior chamber angle was graded with Scheie system by a single experienced ophthalmologist (HYZ). Visual field was assessed using the Humphrey perimetry system (24 − 2 SITA standard test, Humphrey, Carl Zeiss Meditec, CA) if the BCVA was > 20/400. The AL was measured by the Lenstar LS900 (Haag-Streit, USA). A minimum of five consecutive measurements of AL were performed until five reliable readings were acquired with a signal to noise ratio (SNR) greater than 2.0 for each measurement. Demographic information was recorded, which includes age, sex, and history of peripheral iridotomy, history of suspicion of primary angle closure, PACG, and number of glaucoma medications.

### Ultrasound biomicroscopy

Ultrasound biomicroscopy (UBM) (model SW-3200 L; Tianjin Suowei Electronic Technology Co, Ltd., Tianjin, China) was performed at pre-onset, onset and 6-month after settlement of pseudophakic malignant glaucoma. In the control group, the UBM was performed both before and one month after phacoemulsification. In briefly, patients were anesthetized with topical administration of 0.5% proparacaine hydrochloride. The eye cup without the fundus was placed on the conjunctival sac and an appropriate amount of sterile normal saline was added. The UBM probe was used to examine the eye from 12 o’clock clockwise along its entire circumference, and subsequently two-dimensional images of the anterior segment of the eye were recorded.

**Fig. 1:**
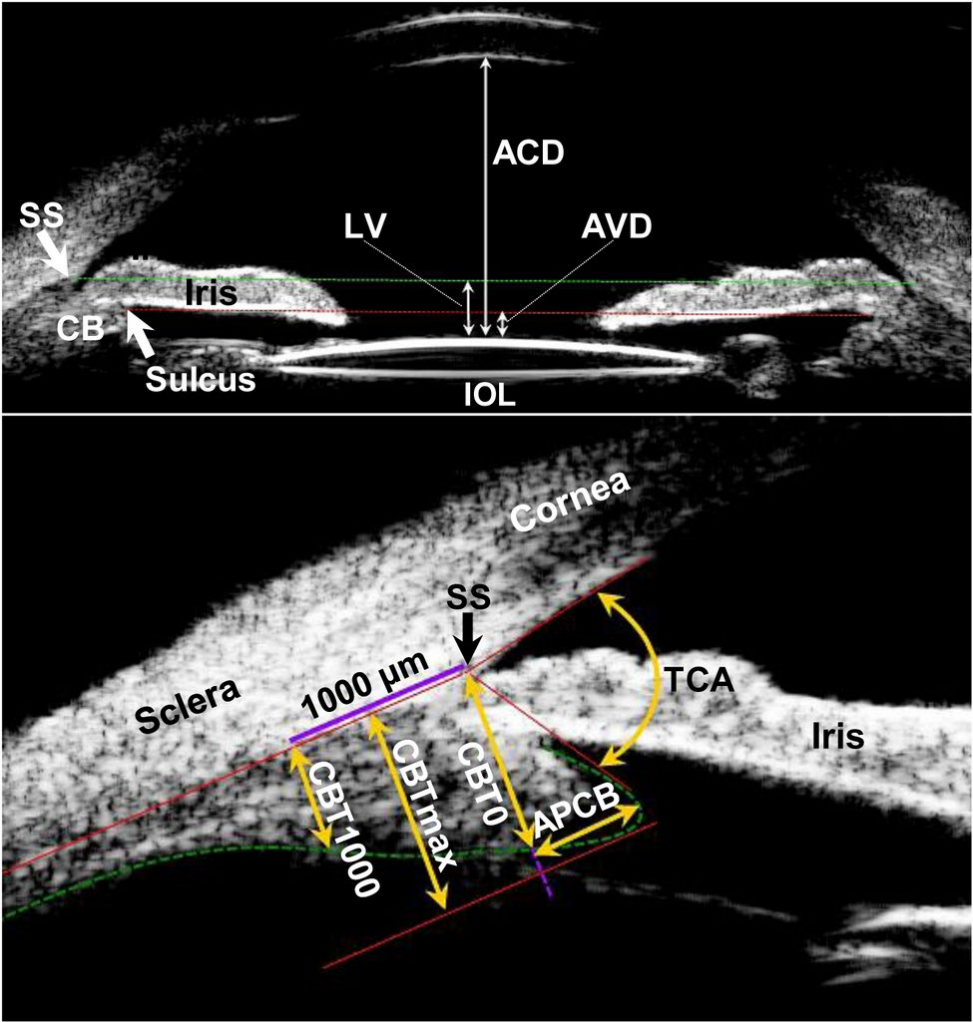
Determination of the following anterior segment parameters by ultrasound biomicroscopy. (**1**) central anterior chamber depth (ACD), the distance between central inner corneal surface and anterior surface of the lens or artificial lens; (**2**) lens vault (LV), the perpendicular distance between the anterior crystalline lens or intraocular lens (IOL) pole and the horizontal line of scleral spurs (SS, green dot line); (**3**) anterior vault distance (AVD), the perpendicular distance between the anterior lens pole and the horizontal line of ciliary sulcus (red dot line); (**4**) maximum ciliary body thickness (CBTmax), the distance from the most inner point of the ciliary body to the inner border of the sclera or its extended line; (**5**) ciliary body thickness at the point of the scleral spur (CBT0) and at a distance of 1000 mm (CBT1000) from the scleral spur; (**6**) anterior placement of the ciliary body (APCB), the maximal distance from the anterior margin of the ciliary body to the vertical line extended from the scleral spur; and (**7**) trabecular-ciliary angle (TCA), the angle between the posterior surface of the cornea and the anterior surface of the ciliary body

After capturing images from axial scans and longitudinal scans, the best images were selected to identify the following parameters at every clock position as described previously ^12^ (Fig. 1): (1) central anterior chamber depth (ACD) - the distance between inner surface of the central cornea and anterior surface of the lens or artificial lens; (2) anterior chamber width (ACW) - the length between the scleral spurs on two sides; (3) lens vault (LV) - the perpendicular distance between the anterior pole of the lens and the horizontal line joining the temporal and nasal scleral spurs; (4) sulcus to sulcus (STS) length; (5) anterior vault distance (AVD) - the perpendicular distance between anterior pole of the lens and the horizontal line connecting the ciliary sulcus on both sides; (6) maximum ciliary body thickness (CBTmax) - the distance from the most inner point of the ciliary body to the inner border of the sclera or its extended line; (7) CBT0 and CBT1000 - the thickness of ciliary body at the point of the scleral spur (CBT0) and at a distance 1000 μm from the scleral spur (CBT1000); (8) anterior placement of the ciliary body (APCB) - the maximal distance from the anterior margin of the ciliary body to the vertical line extended from the scleral spur; and (9) trabecular-ciliary angle (TCA) - the angle between the posterior surface of the cornea and the anterior surface of the ciliary body. The accuracy and repeatability of the ciliary body measurements using UBM have been confirmed previously [12, 14]. The quantitative measurements were performed by three experienced physicians (YLZ, YLC and WJX). Each parameter was measured three times with median recorded and the average from superior, nasal, inferior, and temporal quadrant of the eyeball represented the value for the individual parameter.

### Treatments of malignant glaucoma

Patients with malignant glaucoma were initially treated with non-surgical approaches, including atropine eye ointment (10.0%) and other anti-glaucoma eye drops. If the intraocular pressure did not show significant reduction after 1 to 3 days of conservative medical treatment, and the anterior chamber depth remained shallow or disappeared, surgical zonulo-hyaloido-vitrectomy (ZHV) surgery will be performed immediately as previously described [15]. In brief, with a flow of balanced salt solution into the anterior chamber, a 25 Gauge vitreous cutter was used to establish a pathway between the anterior chamber and the vitreous cavity by zonulectomy, hyaloidectomy, and anterior vitrectomy through the peripheral iris defect. The patients were followed up for 6 months.

### Statistical analysis

Data analysis was conducted using SPSS software version 20.0 (SPSS Inc., Chicago, Illinois, USA). Continuous data were presented as mean ± SD or medians (IQR), while categorical data were shown as numbers. Independent samples t-test and Mann-Whitney U test were used to compare the data with normal distribution and non-normal distribution, respectively. Chi-squared test was used to compare the categorical data. The diagnostic ability of anterior segment parameters on pseudophakic malignant glaucoma was analyzed by receiver operating characteristic curve (ROC). The average of area under the curve (AUC) and the cutoff point with its sensitivity and specificity were reported. A *P* values less than 0.05 was considered as statistically significant.

## Results

### Clinical characteristics of patients

The clinical characteristics of the patients who developed malignant glaucoma after cataract surgery were compared with those without post-operative malignant glaucoma (matched eyes, Table 1). No significant difference was observed in the variables examined, including age, sex, best-corrected visual acuity, intraocular pressure, cup-to disc ratio, degree of angle closure, number of anti-glaucoma eye drops, and axial length between the group with malignant glaucoma and matched eyes.

**Table 1:** Baseline characteristics of the patients with primary angle closure glaucoma (PACG)

Variables	Malignant glaucoma (*n* = 30)	Matched eyes (*n* = 30)	*P* value
Age (yrs)	60.50 (52.75, 69.00)	64.00 (61.75, 72.25)	0.083
Sex (male/female)	10/20	8/22	0.573
BCVA (LogMAR)	0.30 (0.00, 1.00)	0.10 (0.04, 0.40)	0.252
IOP (mmHg)	18.57 ± 4.26	17.10 ± 5.22	0.236
C/D ratio	0.70 (0.50, 0.93)	0.70 (0.40, 0.83)	0.540
Degrees of angle closure	180.00 (150.00, 247.50)	240.00 (120.00, 330.00)	0.676
No. of anti-glaucoma eye drops	3.00 (2.00, 3.00)	2.50 (2.00, 3.00)	0.871
Axial length (mm)	21.96 ± 0.61	22.25 ± 0.71	0.101

BCVA, best-corrected visual acuity; IOP, intraocular pressure; C/D ratio, cup-to-disc ratio. Data are expressed as means ± SD for normal continuous variables, median (Q1, Q3) for non-normal variables or numbers. *P* value was calculated by independent samples t-test, Mann-Whitney U test or Chi-squared test

### Anterior segment parameters at pre-onset and onset of malignant glaucoma

**Fig. 2:**
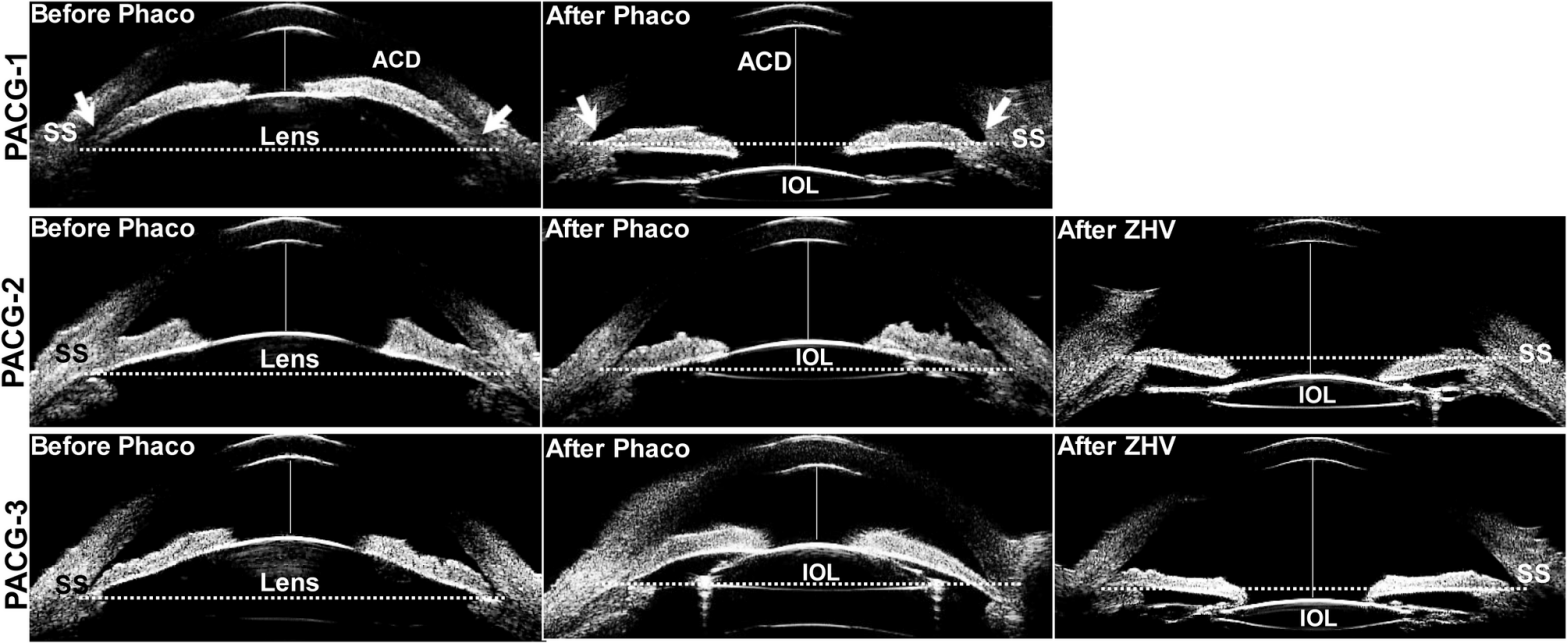
Ultrasound biomicroscopy images of the anterior segments in the patients with primary angle-closure glaucoma (PACG). In a patient without malignant glaucoma indicated as PACG-1, the depth of the anterior chamber (ACD, the distance between central inner corneal surface and anterior surface of the lens or artificial lens) was 1.42 mm with an extremely occluded angle (arrows) before phacoemulsification (Phaco). But the lens extraction with implantation of an artificial intraocular lens (IOL) significantly increased the ACD to 3.50 mm and widened the angle (arrows in After Phaco). A dotted line between the scleral spurs (SS) served as the base line of reference for the position of the IOL. Normally, the IOL was located below the dotted line and the iris tilted toward but without touching the IOL. However, in PACG-2 and PACG-3 with onset of malignant glaucoma, lens extraction did not significantly increase the ACD (2.06 mm before Phaco vs. 2.25 after Phaco in PACG-2, 1.78 mm before Phaco vs. 1.83 after Phaco in PACG-3) and open the angle. The iris of these two patients touched the IOL’s surface and were pushed forward exceeding the dotted line. After zonulo-hyaloido-vitrectomy (ZHV), the iris-IOL diaphragm was shifted backward, substantially deepening the ACD (3.30 mm in PACG-2 and 3.66 mm in PACG-3) and reopening the angle

Quantitative measurements from superior, nasal, inferior and temporal quadrant of the eyeball by using ultrasound biomicroscopy were summarized in Supplemental Table 1. Before the onset of malignant glaucoma, no significant difference was observed in ACD, ACW, LV, STS, AVD, CBT0, CBT1000, APCB and TCA between the group of malignant glaucoma and the matched eyes. Notably, in the malignant glaucoma group, the CBTmax was significantly thinner than that of matched eyes (0.87 ± 0.09 mm vs. 0.95 ± 0.09 mm, *P* = 0.001, as shown in Table 2). However, when compared to fellow eyes, the CBTmax in the malignant glaucoma group exhibited a trend toward thinning, although this difference did not reach statistical significance (*P* = 0.15, as shown in Supplemental Table 2). At the onset of malignant glaucoma, as shown in Fig. 2 (PACG-2 and PACG-3), the iris-IOL diaphragm was displaced forward across the line connecting the scleral spurs on both sides in the UBM images, leading to a marked decrease in ACD (*P* < 0.001) and substantial increase in LV (*P* < 0.001) and AVD (*P* < 0.001), when compared to the matched eyes (Table 2). In malignant glaucoma lens extraction did not improve the CBTmax (0.87 ± 0.09 mm before vs. 0.87 ± 0.11 mm after), CBT0 (0.82 ± 0.09 mm before vs. 0.81 ± 0.10 mm), CBT1000 (0.57 ± 0.06 mm before vs. 0.56 ± 0.07 mm after), and TCA (57.59 ± 17.81 before mm vs. 53.92 ± 13.41 mm after). Interestingly, in the matched eyes cataract surgery increased the CBTmax from 0.95 ± 0.09 mm to 1.05 ± 0.11 mm, CBT0 from 0.85 ± 0.11 mm to 0.92 ± 0.13 mm, CBT1000 from 0.61 ± 0.08 mm to 0.63 ± 0.08 mm and TCA from 62.61 ± 13.17 mm to 70.92 ± 10.65 mm (Table 2).

**Table 2:** Quantitative measurements by ultrasound biomicroscopy (UBM) in recruited eyes before and at the onset of malignant glaucoma

**Parameters**	Before cataract surgery (means from 4 quadrants in UBM)				After cataract surgery (means from 4 quadrants in UBM)		
	Pre-onset of malignant glaucoma	Matched eyes	*P* value		Onset of malignant glaucoma	Matched eyes	*P* value
	(*n* = 30)	(*n* = 30)			(*n* = 30)	(*n* = 30)	
ACD	1.97 ± 0.12	1.99 ± 0.27	0.638		2.41 (2.25, 2.53)	3.54 (3.39, 3.75)	< 0.001*
ACW	10.34 ± 0.43	10.14 ± 0.35	0.058		10.17 ± 0.36	10.26 ± 0.40	0.389
LV	0.65 ± 0.19	0.71 ± 0.09	0.101		0.46 (0.32, 0.58)	-0.60 (-0.75, -0.52)	< 0.001*
STS	9.88 (9.75, 10.08)	9.75 (9.45 10.08)	0.133		9.90 ± 0.35	9.72 ± 0.39	0.069
AVD	1.02 ± 0.09	1.04 ± 0.16	0.531		0.69 (0.54, 0.74)	-0.35 (-0.48, -0.21)	< 0.001*
CBTmax	0.87 ± 0.09	0.95 ± 0.09	0.001*		0.87 ± 0.11	1.05 ± 0.11	< 0.001*
CBT0	0.82 ± 0.09	0.85 ± 0.11	0.347		0.81 ± 0.10	0.92 ± 0.13	< 0.001*
CBT1000	0.57 ± 0.06	0.61 ± 0.08	0.069		0.56 ± 0.07	0.63 ± 0.08	0.001*
APCB	0.45 ± 0.19	0.48 ± 0.19	0.595		0.44 ± 0.14	0.44 ± 0.20	0.923
TCA	57.59 ± 17.81	62.61 ± 13.17	0.220		53.92 ± 13.41	70.92 ± 10.65	< 0.001*

ACD, central anterior chamber depth; ACW, anterior chamber width; LV, lens vault; STS, sulcus to sulcus; AVD = anterior vault distance; CBTmax, maximum ciliary body thickness; CBT0, ciliary body thickness at point of the scleral spur; CBT1000, ciliary body thickness at 1000 mm from the scleral spur; APCB, anterior placement of ciliary body; TCA, trabecular ciliary process angle. Data are expressed as means ± SD for normal continuous variables and median (Q1, Q3) for non-normal variables. The mean in each parameter is the average of superior, nasal, inferior, and temporal quadrant of the eyeball in UBM. *P* value was calculated by independent samples t-test or Mann-Whitney U test. * indicates *P* value less than 0.05

### Anterior segment parameters after settlement of malignant glaucoma

**Fig. 3:**
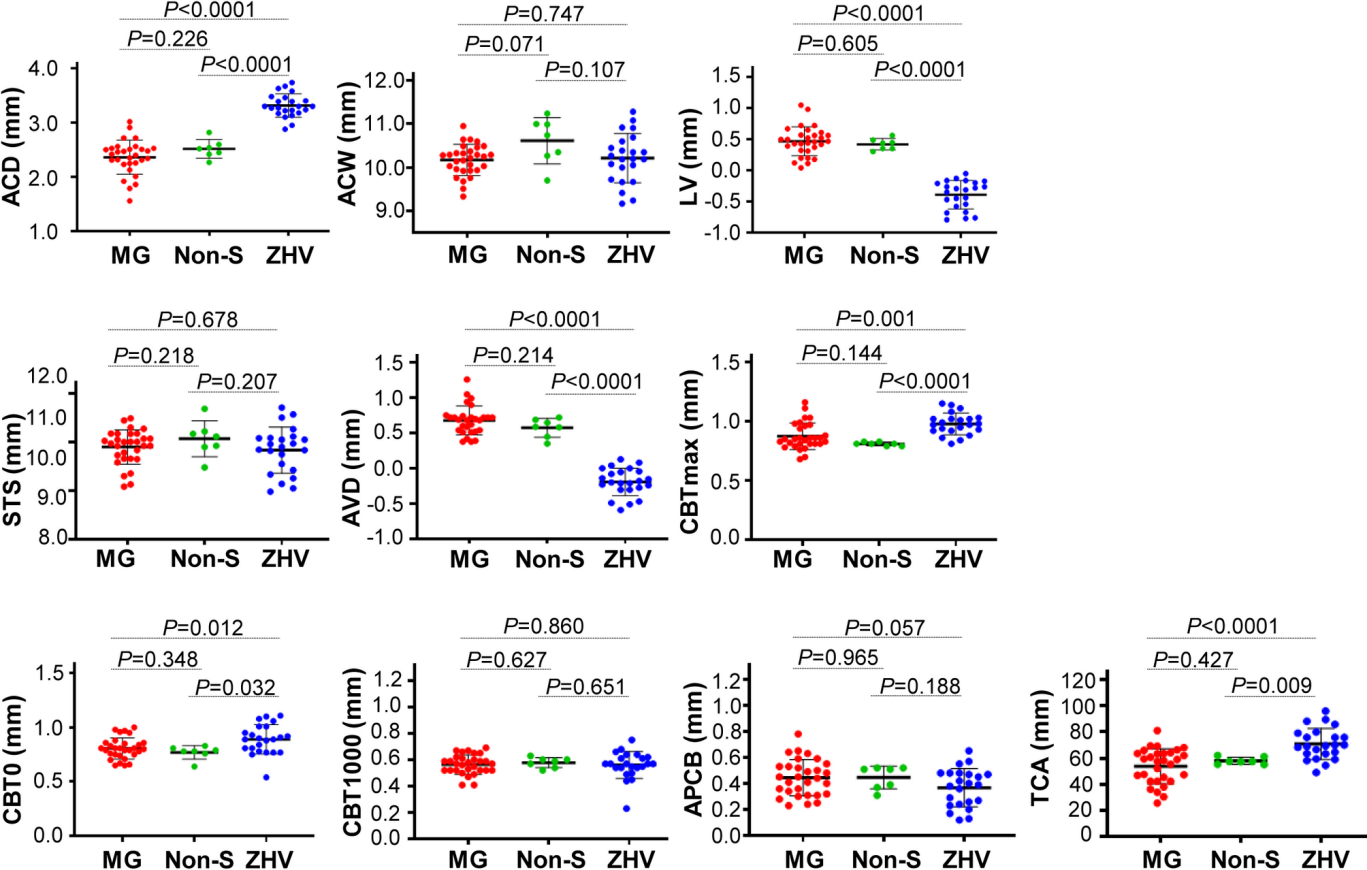
The changes of the anterior segments after treatment with non-surgical approach (Non-S) and surgical zonulo-hyaloido-vitrectomy (ZHV). Treatment with anterior vitrectomy significantly produced an increase in central anterior chamber depth (ACD), maximum ciliary body thickness (CBTmax), ciliary body thickness at point of the scleral spur (CBT0), and trabecular ciliary process angle (TCA) but generated a decrease in the lens vault (LV), anterior vault distance (AVD) when compared to those at the onset of malignant glaucoma (MG) and in the group treated with atropine eye ointment. However, no significant change was observed in these parameters after treatment with atropine eye ointment. ACW, anterior chamber width; LV, lens vault; STS, sulcus to sulcus; AVD, the perpendicular distance between the anterior lens pole and the horizontal line of ciliary sulcus; CBT1000, ciliary body thickness at 1000 mm from the scleral spur; APCB, anterior placement of ciliary body; Data are expressed as means ± SD, The mean in each parameter is the average of superior, nasal, inferior, and temporal quadrant of the eyeball in ultrasound biomicroscopy. *P* value was calculated by independent samples t-test

There were 30 eyes of 30 patients with malignant glaucoma attack. At 6-month after treatment, 7 eyes (23.3%) were resolved by non-surgical approaches, and 23 eyes (76.7%) required surgical zonulo-hyaloido-vitrectomy. As shown in Fig. 3, compared to those before treatment, non-surgical approaches did not cause significant change to the following parameters: ACD, ACW, LV, STS, AVD, CBTmax, CBT0, CBT1000, APCB and TCA. However, surgical zonulo-hyaloido-vitrectomy significantly increased the ACD from 2.36 ± 0.31 mm to 3.32 ± 0.21 mm, CBTmax from 0.87 ± 0.11 mm to 0.96 ± 0.12 mm, CBT0 from 0.81 ± 0.10 mm to 0.89 ± 0.14 mm and TCA from 53.92 ± 13.41 mm to 70.95 ± 11.89 mm, and decreased the LV from 0.47 ± 0.23 mm to -0.39 ± 0.23 mm and AVD from 0.72 ± 0.29 mm to -0.19 ± 0.19 mm. Also, the surgery improved significantly the above parameters when compared with that treated with non-surgical approaches. The representative cases shown in Fig. 2 (PACG-2 and PACG-3) demonstrated that zonulo-hyaloido-vitrectomy caused a backward shift of the iris-IOL diaphragm, substantially deepening the ACD and reopening the anterior chamber angle. Analysis demonstrated no significant differences in parameters between the surgical and non-surgical groups (Supplemental Table 3).

### Diagnostic ability of anterior segment parameters on malignant glaucoma

The ROC analysis of anterior segment parameters in the eyes at pre-onset (model 1) and onset (model 2) of malignant glaucoma versus individual matched eyes was shown in Table 3. In model 1, CBTmax was the only factor producing significantly prognostic value (AUC, 0.74 [95% CI, 0.61–0.87], *P* = 0.001). In model 2, at the onset of malignant glaucoma, the AUC of CBTmax (0.86, [95% CI, 0.76–0.96], *P* < 0.001), CBT0 (0.76, [95% CI, 0.64–0.89], *P* = 0.001) and CBT1000 (0.73, [95% CI, 0.60–0.85], *P* = 0.003) were significantly increased. The ACD, LV, AVD, and TCA, but not ACW, STS and APCB, would become a significant risk factor for malignant glaucoma (Table 3).

**Table 3:** Receiver operating characteristic (ROC) curves of the parameters in anterior segment of the recruited eyes

	AUC	95% CI	*P* value
Model 1. Before cataract surgery Eyes with pre-onset of malignant glaucoma vs. Matched eyes			
ACD	0.51	0.35–0.66	0.923
ACW	0.61	0.47–0.76	0.128
LV	0.58	0.43–0.74	0.268
STS	0.61	0.47–0.76	0.133
AVD	0.52	0.37–0.67	0.756
CBTmax	0.74	0.61–0.87	0.001^*^
CBT0	0.54	0.39–0.69	0.589
CBT1000	0.59	0.45–0.74	0.212
APCB	0.53	0.39–0.68	0.663
TCA	0.59	0.44–0.73	0.237
Model 2. After cataract surgery Eyes with onset of malignant glaucoma vs. Matched eyes			
ACD	1.00	0.99-1.00	< 0.001^*^
ACW	0.57	0.43–0.72	0.322
LV	1.00	1.00–1.00	< 0.001^*^
STS	0.65	0.50–0.79	0.053
AVD	1.00	1.00–1.00	< 0.001^*^
CBTmax	0.86	0.76–0.96	< 0.001^*^
CBT0	0.76	0.64–0.89	0.001^*^
CBT1000	0.73	0.60–0.85	0.003^*^
APCB	0.52	0.36–0.67	0.830
TCA	0.83	0.73–0.93	< 0.001^*^

AUC, area under the curve; CI, confidence interval; ACD, central anterior chamber depth; ACW, anterior chamber width; LV, lens vault; STS, sulcus to sulcus; AVD, the perpendicular distance between the anterior lens pole and the horizontal line of ciliary sulcus; CBTmax, maximum ciliary body thickness; CBT0, ciliary body thickness at point of the scleral spur; CBT1000, ciliary body thickness at 1000 mm from the scleral spur; APCB, anterior placement of ciliary body; TCA, trabecular ciliary process angle; * indicates *P* value less than 0.05

## Discussion

The exact pathophysiologic mechanism of malignant glaucoma is not fully understood. An anteriorly rotated ciliary body was reported previously in UBM images during an episode of malignant glaucoma [16, 17], which was further identified as a larger anterior placement of the ciliary body (APCB) [12]. However, it should be noted that UBM in these studies was performed at the onset of malignant glaucoma, where treatments with anti-glaucoma eye drops often affect the ciliary body [13, 18]. Importantly, the forward movement of iris-lens diaphragm has already occurred, causing the formation of larger APCB. In the current study we reviewed 30 cases of pseudophakic malignant glaucoma, which had UBM images captured before the attack. Unexpectedly, in these eyes we did not find a significant increase in APCB and reduction in TCA, both representing anterior positioning of the ciliary body [19]. These findings suggest that the anterior rotation of ciliary body might not be sufficient to serve an early predictor of malignant glaucoma.

Nevertheless, we found that the affected eyes had a significant reduction in CBTmax. The CBTs, including CBTmax, CBT0 and CBT1000, are the parameters indicating the thickness of the ciliary body [12]. CBTmax represents the thickness of ciliary crown and CBT1000 represents that of pars plana [20]. In the current study, we found no significant difference in CBT0 and CBT1000, but a marked reduction in thickness of the CBTmax at pre-onset of malignant glaucoma. In an earlier study a thinning of ciliary body (CBTmax, 0.55 ± 0.09 mm) was also reported in malignant glaucoma [12], which were even smaller than our measurements (CBTmax, 0.87 ± 0.09 mm). It is possible that the UBM in that study was done during an episode of malignant glaucoma, while we performed it before the event. Furthermore, we found that malignant glaucoma attack did not further decrease the thickness of ciliary body (CBTmax, 0.87 ± 0.11 mm). However, in eyes without occurrence of malignant glaucoma, the CBTmax, CBT0 and CBT1000 were increased from 0.95 ± 0.09 mm, 0.85 ± 0.11 mm and 0.61 ± 0.08 mm to 1.05 ± 0.11 mm, 0.92 ± 0.13 mm and 0.63 ± 0.08 mm after lens extraction, respectively. These findings suggest that cataract surgery increases the thickness of the ciliary body, but such changes are not observed in the eyes with subsequent development of malignant glaucoma, revealing a reduction in thickness of the ciliary body might be a possible predictor for post-operative development of malignant glaucoma. Thinning of the ciliary body was thought to be atrophied, which causes anterior displacement of ciliary body. Since the ciliary body is firmly attached to the scleral spur, it may prevent ciliary processes from anterior rotation [21]. Therefore, at the onset of malignant glaucoma attack, it is reasonable to argue that the affected eyes with smaller ciliary body may result in an increase of APCB and a reduction of TCA. Occurrence of the anteriorly rotated ciliary body is likely to induce angle closure by loosening the lens zonules [21, 22]. The eye with angle closure is more vulnerable to develop postsurgical malignant glaucoma [23]. On the other hand, a thicker ciliary body as observed in the matched eyes maintains the normal position of ciliary processes that contributes to the remission of malignant glaucoma. We thus propose that the thickness of ciliary body plays a crucial role in development of malignant glaucoma, and a reduction in the thickness should be considered as an early predictor.

To substantiate this assumption, the parameters were measured again after settlement of malignant glaucoma. We found no significant difference in all variables examined, including ACD, CBTmax and TCA, after treating with non-surgical approaches, probably due to a small sample size in this group. However, surgical zonulo-hyaloido-vitrectomy increased substantially the ACD and markedly decreased the LV and AVD. At 6-month after the surgery, none of these eyes developed recurrence, which agrees with the reports that zonulo-hyaloido-vitrectomy resolves pseudophakic malignant glaucoma with a 100% success rate [15, 24]. Notably, in these eyes the surgery significantly increased the CBTmax, CBT0 and enlarged the TCA, but did not alter the CBT1000 and APCB. The mechanism on CBTmax improvement by surgery remains unknown. As mentioned above, when CBTmax increases, the ciliary body may attach more strongly to the scleral spur that likely contributes to the expansion of TCA and subsequent alleviation of the malignant glaucoma. Among these parameters, the CBTmax could be considered as the most critical factor for the development of pseudophakic malignant glaucoma, as it becomes thinner during the attack and is thickened when the attack subsides in our cohorts. Moreover, at pre-onset of malignant glaucoma, CBTmax is the only factor that produces significant prognostic value (AUC, 0.74 [95% CI, 0.61–0.87]) for the malignant glaucoma, which almost reaches the predictive ability at its attack (AUC, 0.86 ([95% CI, 0.76–0.96]). These results support strongly that a reduced CBTmax could be a pre-existing predictor for pseudophakic malignant glaucoma.

## Conclusion

This study was limited by the small sample size, but was strengthened by the results obtained before malignant glaucoma attack and follow-up post-surgical assessments. The findings provide the first evidence that a reduced CBTmax may serve as an early predictor for vulnerability to pseudophakic malignant glaucoma. Determination of the CBTmax using UBM is suggested if patient is diagnosed with short AL and still has a shallow ACD after ocular surgery.

## Electronic supplementary material

Below is the link to the electronic supplementary material.

Supplementary Material 1

Supplementary Material 2

Supplementary Material 3

## Data Availability

The datasets generated and analyzed during the current study are available. The materials used in this study are available. Requests for access to the data and materials should be directed to the corresponding author (Hong Yang Zhang, hy3005716@163.com).
