# Supplementary material for: Thinning of maximum ciliary body thickness: a potential early indicator for pseudophakic malignant glaucoma in primary angle closure glaucoma
Source: BMC Ophthalmol. 2025 Apr 28;25:250. doi: 10.1186/s12886-025-04100-0 (PMC12036250; doi:10.1186/s12886-025-04100-0)
Supplement: Supplementary file 1 — Supplementary Material 1 [file 12886_2025_4100_MOESM1_ESM.docx]

|  | Before cataract surgery | |  | After cataract surgery | |  | Settlement of malignant glaucoma | |
| --- | --- | --- | --- | --- | --- | --- | --- | --- |
| Parameters | Pre-onset of malignant glaucoma (n=30) | Matched eyes (n=30) |  | Onset of malignant glaucoma (n=30) | Matched eyes (n=30) |  | Non-surgical approach (n=7) | Zonulo-hyaloido-vitrectomy (n=23) |
| ACD |  |  |  |  |  |  |  |  |
| Superior | 1.97 ± 0.12 | 2.00 ± 0.30 |  | 2.36 ± 0.30 | 3.55 ± 0.29 |  | 2.54 ± 0.18 | 3.31 ± 0.21 |
| Nasal | 1.96 ± 0.13 | 2.01 ± 0.28 |  | 2.37 ± 0.33 | 3.56 ± 0.32 |  | 2.53 ± 0.19 | 3.32 ± 0.23 |
| Inferior | 1.97 ± 0.12 | 2.02 ± 0.28 |  | 2.38 ± 0.33 | 3.56 ± 0.30 |  | 2.52 ± 0.15 | 3.32 ± 0.25 |
| Temporal | 1.97 ± 0.16 | 1.94 ± 0.27 |  | 2.35 ± 0.33 | 3.51 ± 0.31 |  | 2.47 ± 0.20 | 3.31 ± 0.21 |
| ACW |  |  |  |  |  |  |  |  |
| Superior | 10.37 ± 0.44 | 10.24 ± 0.48 |  | 10.29 ± 0.50 | 10.31 ± 0.49 |  | 10.59 ± 0.55 | 10.20 ± 0.60 |
| Nasal | 10.35 ± 0.41 | 10.15 ± 0.41 |  | 10.22 ± 0.43 | 10.27 ± 0.52 |  | 10.62 ± 0.50 | 10.22 ± 0.55 |
| Inferior | 10.33 ± 0.45 | 10.14 ± 0.40 |  | 10.14 ± 0.36 | 10.26 ± 0.43 |  | 10.56 ± 0.50 | 10.22 ± 0.57 |
| Temporal | 10.30 ± 0.46 | 10.05 ± 0.40 |  | 10.03 ± 0.46 | 10.18 ± 0.59 |  | 10.68 ± 0.61 | 10.20 ± 0.57 |
| LV |  |  |  |  |  |  |  |  |
| Superior | 0.66 ± 0.21 | 0.73 ± 0.14 |  | 0.46 ± 0.28 | -0.60 ± 0.21 |  | 0.43 ± 0.08 | -0.36 ± 0.24 |
| Nasal | 0.68 ± 0.20 | 0.70 ± 0.09 |  | 0.47 ± 0.24 | -0.60 ± 0.26 |  | 0.43 ± 0.09 | -0.41 ± 0.23 |
| Inferior | 0.64 ± 0.20 | 0.70 ± 0.11 |  | 0.48± 0.24 | -0.59 ± 0.22 |  | 0.43 ± 0.12 | -0.40 ± 0.22 |
| Temporal | 0.62 ± 0.23 | 0.72 ± 0.14 |  | 0.47 ± 0.24 | -0.61± 0.19 |  | 0.39 ± 0.14 | -0.40 ± 0.24 |
| STS |  |  |  |  |  |  |  |  |
| Superior | 9.92 ± 0.31 | 9.75 ± 0.45 |  | 10.05 ± 0.51 | 9.81 ± 0.41 |  | 10.06 ± 0.36 | 9.83 ± 0.49 |
| Nasal | 9.87 ± 0.34 | 9.70 ± 0.43 |  | 9.98 ± 0.45 | 9.85 ± 0.56 |  | 10.10 ± 0.33 | 9.85 ± 0.47 |
| Inferior | 9.85 ± 0.35 | 9.75 ± 0.36 |  | 9.90 ± 0.34 | 9.66 ± 0.51 |  | 10.08 ± 0.36 | 9.84 ± 0.47 |
| Temporal | 9.86 ± 0.33 | 9.80 ± 0.35 |  | 9.67 ± 0.53 | 9.58 ± 0.55 |  | 10.06 ± 0.46 | 9.83 ± 0.48 |
| AVD |  |  |  |  |  |  |  |  |
| Superior | 1.02 ± 0.10 | 1.05 ± 0.20 |  | 0.72 ± 0.31 | -0.31± 0.20 |  | 0.59 ± 0.12 | -0.18 ± 0.19 |
| Nasal | 1.03 ± 0.10 | 1.04 ± 0.19 |  | 0.72 ± 0.31 | -0.35± 0.25 |  | 0.59 ± 0.13 | -0.18 ± 0.21 |
| Inferior | 1.01 ± 0.12 | 1.04 ± 0.20 |  | 0.71± 0.30 | -0.35 ± 0.21 |  | 0.55 ± 0.16 | -0.20 ± 0.20 |
| Temporal | 1.03 ± 0.10 | 1.04± 0.18 |  | 0.72 ± 0.28 | -0.35± 0.20 |  | 0.57 ± 0.18 | -0.21 ± 0.21 |

Supplemental Table 1. Anterior segment measurements of recruited eyes.

Supplemental Table 1 (continued). Anterior segment measurements of recruited eyes.

|  | Before cataract surgery | |  | After cataract surgery | |  | Settlement of malignant glaucoma | |
| --- | --- | --- | --- | --- | --- | --- | --- | --- |
| Parameters | Pre-onset of malignant glaucoma (n=30) | Matched eyes (n=30) |  | Onset of malignant glaucoma (n=30) | Matched eyes (n=30) |  | Non-surgical approach (n=7) | Zonulo-hyaloido-vitrectomy (n=23) |
| CBTmax |  |  |  |  |  |  |  |  |
| Superior | 0.88 ± 0.12 | 0.94 ± 0.14 |  | 0.86 ± 0.12 | 1.04 ± 0.16 |  | 0.82 ± 0.05 | 0.97 ± 0.12 |
| Nasal | 0.87 ± 0.09 | 0.98± 0.12 |  | 0.89 ± 0.12 | 1.03 ± 0.13 |  | 0.81 ± 0.04 | 0.97 ± 0.13 |
| Inferior | 0.85 ± 0.10 | 0.98 ± 0.12 |  | 0.87 ± 0.14 | 1.08 ± 0.16 |  | 0.82 ± 0.08 | 0.96 ± 0.15 |
| Temporal | 0.87 ± 0.10 | 0.91 ± 0.12 |  | 0.87 ± 0.16 | 1.03 ± 0.15 |  | 0.79 ± 0.11 | 0.95 ± 0.13 |
| CBT0 |  |  |  |  |  |  |  |  |
| Superior | 0.83 ± 0.11 | 0.85 ± 0.15 |  | 0.79 ± 0.12 | 0.93 ± 0.16 |  | 0.79 ± 0.06 | 0.90 ± 0.14 |
| Nasal | 0.82± 0.08 | 0.85 ± 0.15 |  | 0.82 ± 0.11 | 0.90 ± 0.17 |  | 0.77 ± 0.07 | 0.91 ± 0.14 |
| Inferior | 0.83± 0.09 | 0.87 ± 0.13 |  | 0.81 ± 0.14 | 0.95± 0.16 |  | 0.77 ± 0.10 | 0.88 ± 0.16 |
| Temporal | 0.84 ± 0.09 | 0.82 ± 0.13 |  | 0.81 ± 0.13 | 0.91 ± 0.17 |  | 0.75 ± 0.13 | 0.87 ± 0.15 |
| CBT1000 |  |  |  |  |  |  |  |  |
| Superior | 0.58 ± 0.09 | 0.60 ± 0.12 |  | 0.57 ± 0.09 | 0.63 ± 0.11 |  | 0.59 ± 0.06 | 0.57 ± 0.13 |
| Nasal | 0.57 ± 0.07 | 0.61 ± 0.12 |  | 0.57 ± 0.10 | 0.62 ± 0.09 |  | 0.56 ± 0.07 | 0.55 ± 0.10 |
| Inferior | 0.56 ± 0.07 | 0.63 ± 0.09 |  | 0.56 ± 0.12 | 0.64 ± 0.11 |  | 0.56 ± 0.09 | 0.56 ± 0.11 |
| Temporal | 0.58 ± 0.10 | 0.60 ± 0.10 |  | 0.56 ± 0.12 | 0.63 ± 0.12 |  | 0.60 ± 0.10 | 0.57 ± 0.13 |
| APCB |  |  |  |  |  |  |  |  |
| Superior | 0.47 ± 0.20 | 0.51 ± 0.24 |  | 0.43 ± 0.19 | 0.44 ± 0.23 |  | 0.43 ± 0.10 | 0.35 ± 0.14 |
| Nasal | 0.44 ± 0.20 | 0.48 ± 0.23 |  | 0.47 ± 0.20 | 0.45 ± 0.25 |  | 0.42 ± 0.10 | 0.39 ± 0.16 |
| Inferior | 0.45 ± 0.21 | 0.45 ± 0.23 |  | 0.41 ± 0.18 | 0.46 ± 0.29 |  | 0.44 ± 0.09 | 0.40 ± 0.17 |
| Temporal | 0.43 ± 0.19 | 0.47 ± 0.27 |  | 0.46 ± 0.18 | 0.41 ± 0.27 |  | 0.50 ± 0.10 | 0.34 ± 0.15 |
| TCA |  |  |  |  |  |  |  |  |
| Superior | 55.50 ± 18.70 | 59.76 ± 18.22 |  | 54.21 ± 17.42 | 67.87 ± 16.11 |  | 60.16 ± 5.34 | 71.37 ± 12.85 |
| Nasal | 58.25 ± 18.76 | 62.31 ± 14.26 |  | 54.35 ± 18.81 | 71.72 ± 14.22 |  | 54.17 ± 3.46 | 71.08 ± 13.44 |
| Inferior | 57.86 ± 20.03 | 64.97 ± 15.91 |  | 53.43 ± 16.26 | 72.17 ± 15.35 |  | 59.47 ± 5.43 | 72.16 ± 12.11 |
| Temporal | 58.76 ± 18.83 | 63.39 ± 16.23 |  | 53.70 ± 18.40 | 71.93 ± 15.01 |  | 58.43 ± 9.70 | 69.19 ± 13.50 |
