# Supplementary material for: Thinning of maximum ciliary body thickness: a potential early indicator for pseudophakic malignant glaucoma in primary angle closure glaucoma
Source: BMC Ophthalmol. 2025 Apr 28;25:250. doi: 10.1186/s12886-025-04100-0 (PMC12036250; doi:10.1186/s12886-025-04100-0)
Supplement: Supplementary file 2 — Supplementary Material 2 [file 12886_2025_4100_MOESM2_ESM.docx]

Supplemental Table 2. Anterior segment measurements of recruited eyes.

|  | Before cataract surgery | |
| --- | --- | --- |
| Parameters | Pre-onset of malignant glaucoma (n=30) | Fellow eyes (n=30) |
| CBTmax |  |  |
| Superior | 0.88 ± 0.12 | 0.91 ± 0.13 |
| Nasal | 0.87 ± 0.09 | 0.90± 0.10 |
| Inferior | 0.85 ± 0.10 | 0.89 ± 0.10 |
| Temporal | 0.87 ± 0.10 | 0.91 ± 0.11 |
| CBT0 |  |  |
| Superior | 0.83 ± 0.11 | 0.85 ± 0.12 |
| Nasal | 0.82± 0.08 | 0.84 ± 0.09 |
| Inferior | 0.83± 0.09 | 0.85 ± 0.10 |
| Temporal | 0.84 ± 0.09 | 0.85 ± 0.10 |
| CBT1000 |  |  |
| Superior | 0.58 ± 0.09 | 0.59 ± 0.10 |
| Nasal | 0.57 ± 0.07 | 0.58 ± 0.08 |
| Inferior | 0.56 ± 0.07 | 0.60 ± 0.10 |
| Temporal | 0.58 ± 0.10 | 0.60 ± 0.11 |
| APCB |  |  |
| Superior | 0.47 ± 0.20 | 0.47 ± 0.21 |
| Nasal | 0.44 ± 0.20 | 0.44 ± 0.22 |
| Inferior | 0.45 ± 0.21 | 0.42 ± 0.18 |
| Temporal | 0.43 ± 0.19 | 0.42 ± 0.20 |
| TCA |  |  |
| Superior | 55.50 ± 18.70 | 57.15 ± 18.93 |
| Nasal | 58.25 ± 18.76 | 60.01 ± 19.12 |
| Inferior | 57.86 ± 20.03 | 60.57 ± 18.87 |
| Temporal | 58.76 ± 18.83 | 60.56 ± 19.06 |
