# Supplementary material for: Thinning of maximum ciliary body thickness: a potential early indicator for pseudophakic malignant glaucoma in primary angle closure glaucoma
Source: BMC Ophthalmol. 2025 Apr 28;25:250. doi: 10.1186/s12886-025-04100-0 (PMC12036250; doi:10.1186/s12886-025-04100-0)
Supplement: Supplementary file 3 — Supplementary Material 3 [file 12886_2025_4100_MOESM3_ESM.docx]

Supplemental Table 3.Quantitative measurements by ultrasound biomicroscopy (UBM) in malignant glaucoma eyes after cataract surgery

|  | **After cataract surgery**  (means from 4 quadrants in UBM) | | |
| --- | --- | --- | --- |
|  | **Surgical** | **Non-Surgical Group** | ***P***  **value** |
| **Parameters** | (n=23) | (n=7) |  |
| ACD | 2.35±0.36 | 2.40± 0.10 | 0.616 |
| ACW | 10.14±0.36 | 10.25±0.36 | 0.486 |
| LV | 0.45 ± 0.26 | 0.51 ± 0.08 | 0.381 |
| STS | 10.00 (9.73,10.17) | 9.79 (9.36,10.05) | 0.339 |
| AVD | 0.70 (0.51,0.74) | 0.69 (0.68,0.75) | 0.572 |
| CBTmax | 0.87 ± 0.12 | 0.87 ± 0.09 | 0.950 |
| CBT0 | 0.81 ± 0.11 | 0.80 ± 0.08 | 0.809 |
| CBT1000 | 0.57 ± 0.08 | 0.56 ± 0.07 | 0.841 |
| APCB | 0.46 ± 0.15 | 0.40 ± 0.10 | 0.367 |
| TCA | 52.48 ± 12.38 | 58.64 ± 10.19 | 0.242 |

ACD, central anterior chamber depth; ACW, anterior chamber width; LV, lens vault; STS, sulcus to sulcus; AVD = anterior vault distance; CBTmax, maximum ciliary body thickness; CBT0, ciliary body thickness at point of the scleral spur; CBT1000, ciliary body thickness at 1000 mm from the scleral spur; APCB, anterior placement of ciliary body; TCA, trabecular ciliary process angle. Data are expressed as means ± SD for normal continuous variables and median (Q1, Q3) for non-normal variables. The mean in each parameter is the average of superior, nasal, inferior, and temporal quadrant of the eyeball in UBM. *P* value was calculated by independent samples t-test or Mann-Whitney U test. * indicates *P* value less than 0.05.
